# Editorial: Application of noninvasive neuromodulation in cognitive rehabilitation, volume II

**DOI:** 10.3389/fneur.2024.1520727

**Published:** 2024-12-16

**Authors:** Pengxu Wei, Le Li, Mariagiovanna Cantone, Ping Gu

**Affiliations:** ^1^Alzheimer's Disease and Cognitive Rehabilitation Committee, Chinese Association of Rehabilitative Medicine, Beijing, China; ^2^Institute of Medical Research, Northwestern Polytechnical University, Xi'an, China; ^3^Neurology Unit, Policlinico University Hospital “G. Rodolico-San Marco”, Catania, Italy; ^4^Department of Neurology, The First Hospital of Hebei Medical University, Hebei, China

**Keywords:** cognitive impairment, cognitive training, rehabilitation, noninvasive neuromodulation, neurological diseases, cerebellum, brainstem

When cognitive dysfunction occurs due to neurological disorders, non-invasive neuromodulation is an effective intervention, if applied properly. This Research Topic aimed to provide a platform for research and discussions in this area.

Wang et al. analyzed the impacts of repetitive transcranial magnetic stimulation (rTMS) on the executive function of patients with vascular cognitive impairment (VCI) in a systematic review and meta-analysis. Their results showed that rTMS could improve executive function in patients with VCI and several modalities/parameters of rTMS, e.g., intermittent theta burst stimulation (iTBS), higher frequency, lower intensity, longer duration, and combined comprehensive therapy could lead to better effects.

Through examining methodological and reporting quality, Zhang et al. assessed the quality of meta-analyses and systematic reviews on rTMS (published before 26/3/2024) for post-stroke cognitive impairment. The authors used radar plots for visualization and found that most assessed literature supported the effectiveness of rTMS, although the overall quality of the literature was low. Similarly, Georgiou stated that it is difficult to draw definitive conclusions and make recommendations for rTMS application on post-stroke aphasia and dysphagia since high-level evidence supporting rTMS effects on post-stroke aphasia/dysphagia remains insufficient. Additionally, the heterogeneity in rTMS methodologies, outcome measures, and the lack of consensus make it difficult to synthesize evidence and develop standardized protocols. In a national survey performed in South Korea, Yu et al. found that 44.3% of hospitals either lacked a treatment protocol or physiatrists were not familiar with the protocol if it existed. Among open-ended responses regarding unmet needs for rTMS therapy, the most common answers included a lack of protocols, guidelines, and education. Taken together, although a growing body of research supports the effects of rTMS, standardized study and application of rTMS remains a challenge.

Additionally, in this Research Topic, Bin-Alamer et al. provided evidence and possible mechanisms by which hyperbaric oxygen therapy improves cognitive function, e.g., mitochondrial function enhancement, neurogenesis, angiogenesis, synaptic and axonal formation, telomere elongation, and anti-inflammation. Kim et al. introduced the protocol of a study applying acupuncture, a minimally invasive method, to treat mild cognitive impairment (MCI). The study will be a randomized, prospective, and active-controlled trial. Compared with previously reported studies applying acupuncture for MCI, this more rigorously designed clinical study is expected to provide additional evidence regarding the efficacy of acupuncture for MCI.

A brief period (4 weeks) of non-invasive neuromodulation combined with language and cognitive training can improve language and cognitive function more than language and cognitive training alone. For instance, Zhou et al. found that transcranial direct current stimulation (tDCS) combined with language and cognitive training improved language and cognitive ability in children with language delay. Notably, the combination therapy is effective in a relatively short period (4 weeks), which is shorter than previously reported durations (6 weeks to 3 months). Additionally, Gan et al. found that low-frequency rTMS combined with speech and language therapy could improve language function in subacute stroke patients with Broca's aphasia. Interestingly, delayed effects were observed 3 months after the end of the 4-week treatment, especially in naming gains. For the first time, the author reported that rTMS could improve language ability and cognitive function in patients with Broca's aphasia in the subacute phase. The results of both studies demonstrate that a combination of multiple types of therapies is superior to monotherapy.

The way tDCS stimulates the brain differs from TMS. For instance, when two electrodes of tDCS are positioned above the right eye and the left motor cortex, the direction of the generated electric field passes all brain tissues between the two electrodes (1). A conventional TMS coil, e.g., a circular or figure-of-eight coil, provides restricted stimulation to superficial cortical targets, typically 2–3 cm in depth (2), and activates cortical neurons in the gyral crown or lip of the sulcus or slightly deeper [“lib” indicates the narrow area above the fundus of sulci, see Figure 3 in Cruz-Rizzolo et al. (3)]. Through the connection between the stimulation site and the distal brain area, TMS is also able to modulate the functional status of brain regions away from the stimulation area (4). For instance, TMS stimulation over the cerebellum may result in motor cortex inhibition through the dentato-thalamo-cortical pathway, which may be used to discriminate progressive supranuclear palsy from other neurodegenerative disorders, e.g., Alzheimer's disease, frontotemporal dementia, and dementia with Lewy bodies (5). Recently, cerebellar activity-targeted non-invasive brain stimulation techniques, e.g., TMS and tDCS, have been applied to tune dysfunctional circuitry in the brain (6).

TMS with a double cone coil (consisting of two loops, the planes of which are at an angle of about 110°) placed over the inion or below it on the median line can directly activate the brainstem at the foramen magnum level (just below the pyramidal decussation) (7). The brainstem is affected early in Alzheimer's disease, e.g., tau pathology occurs in the brainstem before the transentorhinal cortex during the development of Alzheimer's disease (8). This is due to at least two reasons. Firstly, the brainstem is a transit station through which Aβ and tau fibrils propagate from the gut into the brain via the vagus nerve (9). Secondly, blood-brain barrier disruption causes harmful blood components to enter the brain, which correlates with the early onset and progression of many neurological disorders such as Alzheimer's disease (10). Nevertheless, some brainstem midline tissues physiologically/anatomically lack a blood-brain barrier (11) and thus are susceptible to harmful blood components. Hence, neuromodulation of the brainstem, directly (7) or indirectly (12), may have a therapeutic effect on early Alzheimer's disease and other neurological disorders with brainstem involvement. However, few studies have focused on neuromodulation techniques that act on the brainstem.

We look forward to seeing new concepts, insights, and studies to explore and validate non-invasive neuromodulation therapies that are beneficial in improving cognitive impairment and intervening in pathogenesis as well as slowing the progression of some neurodegenerative diseases.
